# Acupressure: a possible therapeutic strategy for anxiety related to COVID-19: a meta-analysis of randomized controlled trials

**DOI:** 10.3389/fmed.2024.1341072

**Published:** 2024-03-21

**Authors:** Zhihua Peng, Yulin Zheng, Zeyu Yang, Hongxiao Zhang, Zhennan Li, Mingzhu Xu, Shaoyang Cui, Run Lin

**Affiliations:** ^1^Shenzhen Hospital of Guangzhou University of Chinese Medicine (Futian), Shenzhen, China; ^2^Guangzhou University of Chinese Medicine, Guangzhou, China; ^3^Shenzhen Polytechnic University, Shenzhen, China; ^4^Department of Rehabilitation Medicine, Shenzhen Hospital, Southern Medical University, Shenzhen, China

**Keywords:** COVID-19, acupuncture, acupressure, anxiety, meta-analysis

## Abstract

**Background:**

From the end of 2019 to December 2023, the world grappled with the COVID-19 pandemic. The scope and ultimate repercussions of the pandemic on global health and well-being remained uncertain, ushering in a wave of fear, anxiety, and worry. This resulted in many individuals succumbing to fear and despair. Acupoint massage emerged as a safe and effective alternative therapy for anxiety relief. However, its efficacy was yet to be extensively backed by evidence-based medicine. This study aimed to enhance the clinical effectiveness of acupoint massage and extend its benefits to a wider population. It undertakes a systematic review of the existing randomized controlled trials (RCTs) assessing the impact of acupoint massage on anxiety treatment, discussing its potential benefits and implications. This research aims to furnish robust evidence supporting anxiety treatment strategies for patients afflicted with COVID-19 disease and spark new approaches to anxiety management.

**Objectives:**

This study evaluates the evidence derived from randomised controlled trials (RCTs), quantifies the impact of acupressure on anxiety manifestations within the general population, and proposes viable supplementary intervention strategies for managing COVID-19 related anxiety.

**Materials and methods:**

This review included RCTs published between February 2014 and July 2023, that compared the effects of acupressure with sham control in alleviating anxiety symptomatology as the outcome measure. The studies were sourced from the multiple databases, including CINAHL, EBM Reviews, Embase, Medline, PsycINFO, Scopus and Web of Science. A meta-analysis was performed on the eligible studies, and an overall effect size was computed specifically for the anxiety outcome. The Cochrane Collaboration Bias Risk Assessment Tool (RevMan V5.4) was employed to assess bias risk, data integration, meta-analysis, and subgroup analysis. The mean difference, standard mean deviation, and binary data were used to represent continuous outcomes.

**Results:**

Of 1,110 studies of potential relevance, 39 met the criteria for inclusion in the meta-analysis. The majority of the studies reported a positive effect of acupressure in assuaging anticipatory anxiety about treatment. Eighteen studies were evaluated using the STAI scale. The acupressure procedures were thoroughly documented, and studies exhibited a low risk of bias. The cumulative results of the 18 trials showcased a more substantial reduction in anxiety in the acupressure group compared to controls (SMD = −5.39, 95% CI −5.61 to −5.17, *p* < 0.01). A subsequent subgroup analysis, based on different interventions in the control group, demonstrated improvement in anxiety levels with sham acupressure in improving changes in anxiety levels (SMD −1.61, 95% CI: −2.34 to −0.87, *p* < 0.0001), and blank controls (SMD −0.92, 95% CI: −2.37 to 0.53, *p* = 0.22).

**Conclusion:**

In the clinical research of traditional Chinese medicine treatment of anxiety, acupressure demonstrated effectiveness in providing instant relief from anxiety related to multiple diseases with a medium effect size. Considering the increasing incidence of anxiety caused by long COVID, the widespread application of acupressure appears feasible. However, the results were inconsistent regarding improvements on physiological indicators, calling for more stringent reporting procedures, including allocation concealment, to solidify the findings.

## Introduction

The widespread impact of the COVID-19 pandemic has brought a myriad of challenges which can be stressful and overwhelming, causing psychological distress requiring urgent interventions. As per a World Health Organization survey, over 93% of countries worldwide reported increased demand mental health services (1). A survey revealed that over 40% respondents reported experiencing at least one adverse mental health condition, including anxiety during the COVID-19 pandemic—United States (2). Anxiety, a common affliction during this pandemic, affects everyone from frontline worker to individuals in nursing centers. While medications including benzodiazepines can address anxiety, they often present undesirable side effects. Therefore, the exploration of alternative, effective treatments to alleviate anxiety is of crucial clinical importance. Acupoint massage, a non-drug treatment based on traditional Chinese medicine, offers a promising solution. The technique of pressing acupoints with fingers or non-invasive tools is simple to operate and is not limited by external factors such as equipment and location. It is especially promising. Many scholars have reported that acupoint massage is safe and effective in relieving various mental and physical diseases. In our belief that the acupoint massage could be used widely in clinical treatment, this will eventually benefit people worldwide. Its simplicity and independence from extensive equipment make it convenient and universally applicable. Numerous studies have reported the safety and effectiveness of acupoint massage in mitigating various mental and physical conditions. This study aims to conduct a systematic review of acupuncture massage’s efficacy in treating anxiety, analyzing its value and advantages, especially during the current global long COVID, and provided solid evidence to formulate effective anxiety-related treatments.

## Materials and methods

### Study search

Electronic medical databases, including CINAHL, EBM Reviews, Embase, Medline, PsycINFO, Scopus and Web of Science were explored to gather clinical studies investigating acupuncture’s impact on anxiety management, with changes in anxiety symptoms as the primary outcomes. The keywords used in each database were (anxi* or nervous* or worry or worried or uneas* or apprehensi* or fret* or angst* or fear* or disquiet* or distress* or stress* or strain*) AND (acupressure or chih-ya or shiatsu or shiatzu or zhi-ya or finger-massage or finger-pressure or Tui-Na). All capturing studies published between February 2014 and July 2023.

### Study selection

Inclusion criteria were formulated using the PICO (Population, Intervention, Context and Outcome) tool (3). The inclusion criteria in this review were as follows:

1. Study design: clinical studies such as case report, case series, case-control study, nonrandomized controlled trial, and randomized controlled trial (RCT).
2. Used acupressure as the sole intervention compared with the control condition of either sham control or standard control (e.g., education).
3. Population and area: no limitation.
4. Grouping: intervention: acupressure; comparison: no limitation.
5. Outcome: both qualitative and quantitative outcomes, including the Hamilton Anxiety Rating Scale and the State-Trait Anxiety Inventory (STAI) were used to assess anxiety severity.

Studies like animal mechanism endeavors, case reports, self-controlled, non-RCTs, random crossover studies, and quasi-randomized trials were excluded.

### Data extraction

Two researchers independently extracted data from the included studies using pre-arranged standardized forms. Extracted data included author information, study designs, sample size, average age of participants, interventions, treatment periods, acupressure points used, experimental and control intervention regimens, outcome measures results, and adverse events. The primary outcome for this review was defined as the change in anxiety level before and immediately after the intervention, evaluated by various scales such as the Visual Analogue Scale for Anxiety (VAS-A), State-Trait Anxiety Inventory (STAI), and many others. Secondary outcomes encompassed measurements such as blood pressure, heart rate, blood oxygen, The Modified Yale Preoperative Anxiety Scale (MYPAS), GAD-7, Quality of Life, and others. Two researchers independently reviewed the searched articles and selected relevant studies, with disagreement resolved through discussions among the research team.

### Data synthesis and statistical analysis

Meta-analysis was conducted only on studies that demonstrated similar clinical characteristics and had no domain rated as high risk according to the Cochrane risk of bias assessment. Heterogeneity among studies was evaluated by calculating the *I*^2^ statistic and *χ*^2^ test (assessing the *p*-value) using Review Manager 5 (V.5.4, The Nordic Cochrane Centre, Copenhagen). Significant heterogeneity was considered when the *p*-value was <0.10 and *I*^2^ > 50%, whereupon a random-effects model was employed for data synthesis. The standardised mean differences (SMDs) with 95% CIs were used for continuous outcomes. The overall effect size was calculated based on the pooled SMD, with Cohen’s categories—0.20, 0.50 and 0.80—interpreted as small, medium and large effects, respectively (4).

### Quality assessment

The methodological quality of identified studies was also assessed according to the quality domains in the Cochrane risk of bias tool. It was used to evaluate the following:

1. Random sequence generation.
2. Allocation concealment.
3. Blinding of participants and personnel.
4. Blinding of outcome assessment incomplete outcome data.
5. Selective reporting.
6. Any other sources of bias.

Each domain was rated as “high” (seriously weakens confidence in the results), “unclear” or “low” (unlikely to seriously alter the result). Given the difficulties in blinding the personnel administering acupressure, we only assessed only the blinding of participants and outcome assessments. To follow the guidelines recommended by the Cochrane Back Review Group, a compliance threshold of <50% of the criteria was associated with bias (5). Studies meeting at least four domains without serious flaws were deemed to have a low risk of bias. Disagreements were resolved by discussion or by a third reviewer (HWHT). Where necessary, attempts were made to contact authors for additional information.

## Results

A total of 2,652 articles were initially identified. Afterward, 1,542 duplicates were excluded, and the remaining 1,110 underwent title and abstract review. In this step, 382 irrelevant articles were removed, leaving 103 full-text articles for review. Sixty-three articles were excluded due to unavailability of full text, 10 due to unclear data, 3 non-Chinese or non-English articles were removed, and 5 non-randomized controlled trials (RCTs) were also excluded. Finally, 39 RCTs (6–44) were included in this review (Table 1).

**Table 1:** Characteristics of included clinical trials.

Studies	Year	Design	Treatment type	Treatment intervention and treatment Session	Control/placebo	Main outcome
Hmwe et al. (6)	2014	RCT	Acupressure	EX-HN3, HT7, KI3;3 min light massage + EX-HN3, HT7 non-fistula hand, and KI3 left and right legs, 3 min each acupoint. 3 sessions/week 4 consecutive weeks	Usual care	DASS-21, GHQ-28
Beikmoradi et al. (7)	2015	RCT	Acupressure	HT7, LI4, LI10, H7, Lu9, DU20, Ren6, EX-HN3, UB13 25–30 min (1 session/day, 10 days) 2 min/acupoint	Fake acupoints Routine care	STAI
Aygin and Şen (8)	2019	RCT	Acupressure	Order: HT7, P6, GB20, ST6 2 min/acupoints 16 min once/day 3 days + standard care	Standard care	VAS-A
Rani et al. (9)	2020	RCT	Acupressure	ST34, ST35, ST36, SP9, SP10, GB34 3 min message around acupoints 12 min acupoints (2 min for each) 2 times/day, 5 days/week	Pharmacological treatment	VAS, DASS-21
Bastani (10)	2016	RCT	Acupressure	P7 3 days on forearms bilaterally within 2 days. Thumb pressure 3–5 kg scale. 3 times/day 9 min for each forearm	Pressure at a sham point	MAQ, VAS-A
Abadi et al. (11)	2018	RCT	Acupressure	HE-7, EX-HN3 5 min	A sham point was pressed for 5 min	STAI
Zick et al. (12)	2018	RCT	Acupressure	(1) Relaxing acupressure, EX-HN3, Anmian, HT7, SP6, LR3. (2) Stimulating acupressure, Du20, RN5, LI4, ST36, SP6, and KI3 daily for 6 weeks	Usual care	HADS
Mohaddes Ardabili et al. (13)	2014	RCT	Hand massage	20 min (10 min for each hand)	—	BSPAS
Dehghanmehr et al. (14)	2019	RCT	Acupressure	Acupressure group: P6 3 days/week 4 weeks, 3–4 kg 8 min Reflexology group: solar network point 3times/week 4 weeks, pressure of 3–4 kg 10 min	Routine treatment	STAI
Pouy et al. (15)	2019	RCT	Acupressure	YT deep massage and clockwise rotation for about 5 min	Sham point superficial massage	STAI
Samadi et al. (16)	2018	RCT	Acupressure	SP 6 acupoint for 30 min	Touch group: Spleen 6 acupoint for 30 min routine care group	FAS
Horiuchi et al. (17)	2014	RCT	Acupressure	GB12, SI17, and LI18 for 5 s 5 sessions thrice/day (on waking, after lunch, and before going to bed) HE-7 each point was heated and massaged for 60 s	Usual	POMS-J
Kanza Gul et al. (18)	2020	RCT	Acupressure	Pressure each point 120 s. 30 s rest, repeated 10 min before the surgery	Hospital protocol + no sedatives	STAI
Vasokolaei et al. (19)	2019	RCT	Acupressure	Acupressure group: P6 10 min/hand Hand reflexology group: massage hands for 10 min/hand	Placebo group: conditions similar to the intervention groups were created, a touch on thumbs	STAI
Mansoorzadeh et al. (20)	2014	RCT	Acupressure	Plastic bead on HT7 point and nondominant ear and pressed those areas with fingers for 10 min. At the same time, pressed the third eye point with the thumb using rotary moves with an average 20–25 times/min for 10 min	Pseudo points including outer corner of the left eyebrow and the beginning of the non-dominant ear cavity	VAS
Genc et al. (21)	2015	RCT	Antiemetic drug + acupressure band	P6 point on both wrists 5 days, taking it off only to wash their hands and arms or to take a shower	Antiemetic drug only	BAI
Mącznik et al. (40)	2017	RCT	Acupressure	Acupressure: LI4 3 min Sham acupressure: a nonactive point 3 min	No acupressure	VAS
Sharifi Rizi et al. (22)	2017	RCT	Acupressure	EX-HN3 and HE7 5 min before surgery	Sham acupoint	STAI, VAS
Rarani et al. (23)	2020	RCT	Acupressure	LI4 and HT7 2 min Sham pressure was used in the placebo group: sham pressure points	No intervention	STAI
Dharwal et al. (41)	2020	RCT	Acupressure	P6 group, LI4 group 3 times 10 min at 30 min intervals	Sham acupoint	DASS-42
Avisa et al. (24)	2018	RCT	Acupressure	5 min for deep breathing exercise and 25 min for acupressure, (5 min for each area) Five areas starts from, i.e., toe of both foot followed by midway between the medial ends of the eyebrow, at the ulnar end of the transverse crease of wrist, at the midway between the tip of the medial malleolus on both legs and two points on the both sole of the foot, i.e., one point for each foot	No	MCDAS
Borji et al. (25)	2019	RCT	Massage	Non aromatic oil about 10–15 min once a day for 20 min for 3 consecutive days	Stay at bed	mYPAS
Kuo et al. (26)	2016	RCT	Acupressure	Acupressure (Group 1): EX-HN3, HT7 acupressure beads 10 min sham (Group 2)	No	STAI
Kafaei-Atrian et al. (27)	2021	RCT	Acupressure	EX-HN3 3–4 kg pressure. 15 min sham group, a sham acupoint	No	STAI
Moradi et al. (28)	2014	RCT	Acupressure	GB21 20 min SP6 20 min	Touched	SAQ
Tseng et al. (29)	2020	RCT	Auricular acupressure	Patches with magnetic beads auricular HT7 14 days	Blank patches	GDS、BAI
Lin et al. (30)	2019	RCT	Auricular acupressure	(SV) the lung, Shenmen, subcortex, liver and spleen,4–6 times/session, 5 sessions/day (morning, after each meal, before bedtime). Replace the SV tape every 3 days. magnetic beads	Routine care	SAS
Luo et al. (31)	2016	RCT	Auricular acupressure	Sham Acupressure: adhesive plaster AA: magnetic ball “relaxation point” 30 min	—	STAI
Bang et al. (32)	2020	RCT	Auricular acupressure	AA (Shenmen, sympathy, occiput, heart, and anterior lobe) for 2 weeks	AA (helix 1, 2, 3, 4, and jaw)	STAI
Sangani et al. (33)	2023	RCT	Acupressure	Acupressure group: the Yin Tang and HT7 points, the sham group: the CV24 and TB5 sham points. Lasted for 30 consecutive days	Sham points	DASS
Lee et al. (42)	2023	RCT	Auricular acupressure	Experimental group: auricular acupressure at the Shenmen point and endocrine point bilaterally	Sham points	The Korean version of the Revised Test anxiety scale and state-trait anxiety levels
Abd Elgwad Ali et al. (34)	2022	RCT	Acupressure massage	Bilateral pressure was applied on the organs at the LI4 point and PC-6 point, for 8 to 20 min in 10 s pressure and 2 s resting periods for each point	No intervention	STAI
Bal et al. (35)	2023	RCT	Acupressure	Heart meridian 7 (HT7), large intestine meridian 4 (LI4), and pericardium meridian (PC6) for a period of 16 min	Sham points and standard treatment	STAI, VAS
Derya Ister et al. (36)	2022	RCT	Acupressure	Hegu, Shenmen, and Yintang acupoints 11 min	No intervention	STAI, VAS
Cai et al. (37)	2022	RCT	Auricular acupressure	Shenmen, subcortex, liver and endocrine 1 min 5 times a day for 14 days change every3 days	Irrelevant auricular points	SAS
Masoudi et al. (38)	2022	RCT	acupressure	Pressure was applied on BL32 acupoint at 3–4 and 7–8 cm dilatations	No intervention	Spielberger
Cho et al. (39)	2021	RCT	Meridian acupressure	GV 20, GB 12, GB 21, LI 11, SI 3, KI 1 2 min 30 s (10 times for 15 s at a time)	No intervention	State Anxiety Inventory scale in Korean
Consolação Soares et al. (43)	2022	RCT	Acupressure	EX-HN3, Shen Men of auricular acupuncture	No acupressure	VPTm
Yanik et al. (44)	2022	RCT	Acupressure	LI4, HT7, and EX-HN3 three times a week for 4 weeks	No acupressure	STAI

### Study characteristics

This Meta-analysis included 39 RCT articles and a total of 3,395 cases, with 1902 and 1,493 cases in the test and control groups, respectively. The study participants mainly consisted of two categories: healthy individuals and patients. The healthy group included women in labor, parents of children undergoing surgery, military personnel, college students and so on. The patient group included pre-and post-surgical patients, cancer survivors, hemodialysis patients, burns, sports injuries and so on. Anxiety was evaluated using several indicators, including STAI, VAS-A, DASS-42, DASS-21, MAQ, HADS, BSPAS, FAS, POMS-J, Beck Anxiety, BAI, MCDAS, mYPAS, SAQ, GAD-7, PQOL, SAS, GDS, and various physiological parameters. Of these, the STAI and VAS were the most commonly applied (see Table 1).

### Quality critical appraisal

Twenty-four (6–9, 13, 16, 19, 22–25, 29–32, 44) of the included RCTs were evaluated as having a low risk of bias in the randomization sequence generation, based on detailed description of randomization methods. Sixteen trials (10–12, 14, 15, 17, 18, 20, 21, 26–28, 33–36, 38, 40, 42) lack detailed information, resulting in an unclear risk of bias in randomization. Four trials (6, 15, 17, 40) used open randomization of random numbers table, resulting in a high risk of bias in concealment. Twelve trials (9, 11, 13, 20–23, 28, 33, 39, 41, 43) without sufficient detail were regarded as having an unclear risk of bias in allocation concealment. The remaining trials were evaluated as having a low risk of bias in allocation concealment.

**Figure 1:**
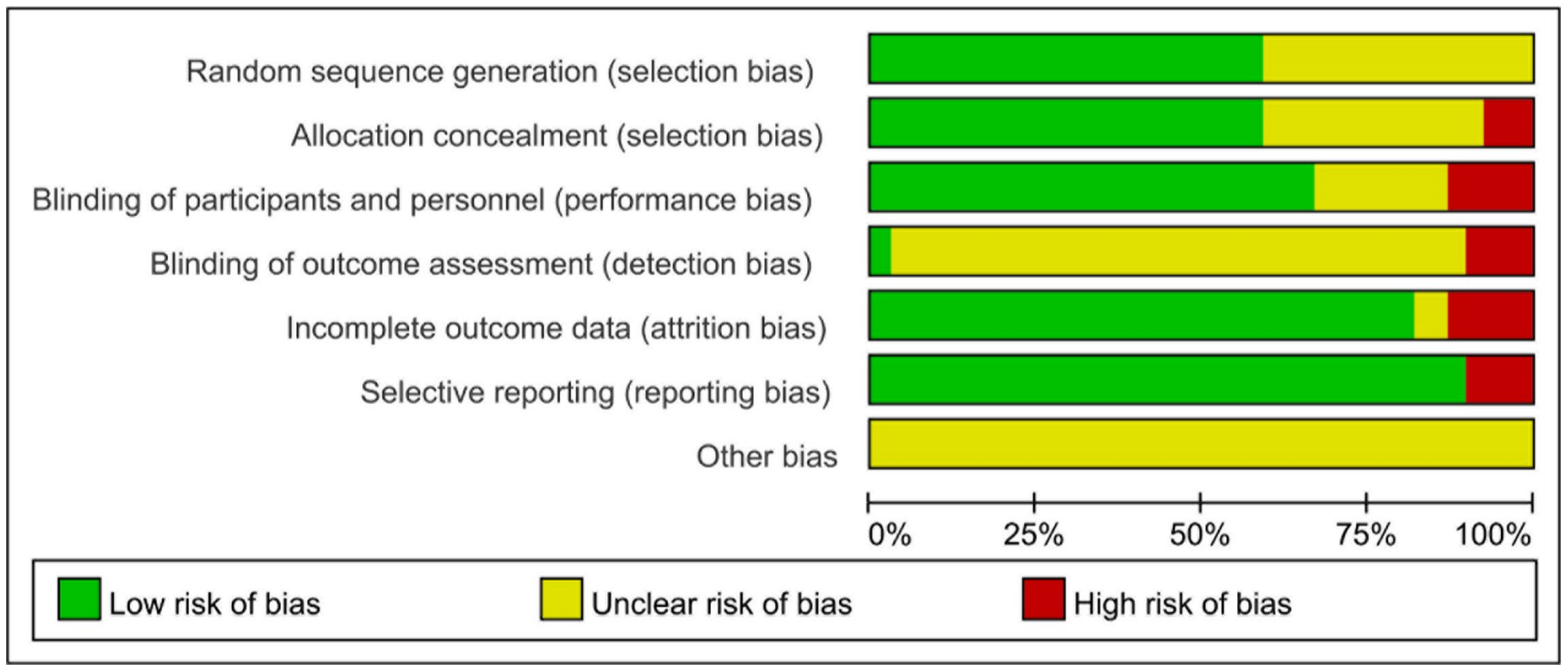
Risk of bias map 1.

**Figure 2:**
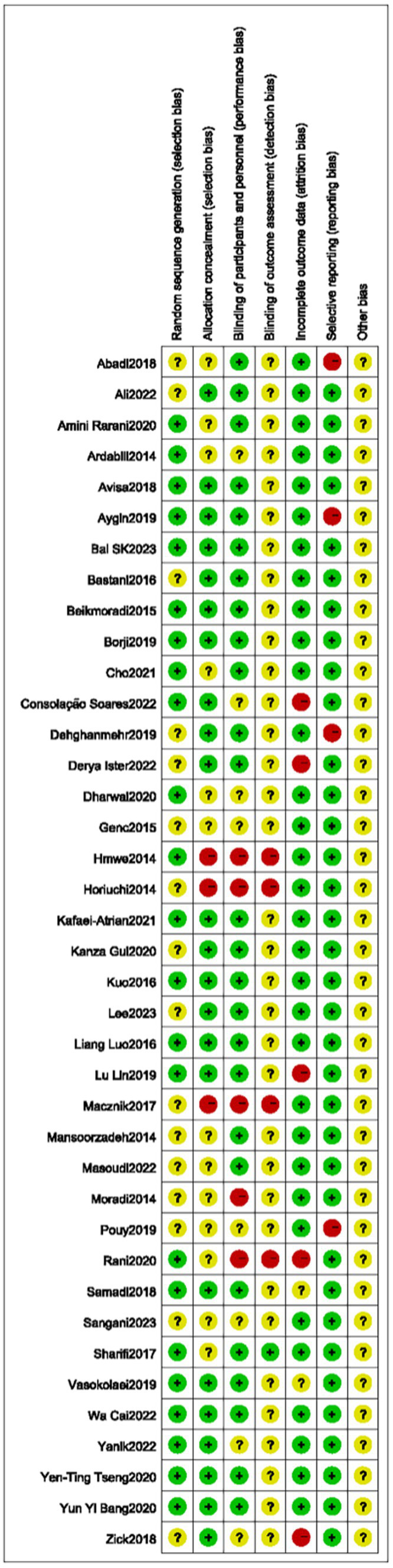
Risk of bias map 2.

Only one (6) of the 39 studies described the blinding method for outcome evaluators. Eight studies (12, 13, 15, 21, 33, 41, 44, 45) did not describe blinding methods, and five (6, 9, 17, 22, 30) indicated that assessors were not blinded. Six studies (9, 12, 16, 30, 36, 43) exhibited a high risk of data integrity. One trial (19) without sufficient detail was considered as having an unclear risk of bias in allocation concealment. Four studies (8, 11, 14, 15) selectively report results, indicating in a high risk of bias. Other studies (20, 23, 36–38, 40) had registered online with specified outcomes, leading to a low risk of bias in selective reporting. All trials had an unknown risk in other sources of bias. Details of the risk of bias were summarized in Table 2, (Figures 1, 2).

**Table 2:** Risk of bias summary for the included studies.

	Random sequence generation	Allocation hiding	Participant and implementer blinding	Incomplete ending data	Selective publication	Other bias
Hmwe	Low risk	High risk	High risk	Low risk	Low risk	Unknown
Beikmoradi	Low risk	Low risk	Low risk	Low risk	Low risk	Unknown
Aygin	Low risk	Low risk	Low risk	Low risk	High risk	Unknown
Rani	Low risk	Unclear	High risk	High risk	Low risk	Unknown
Bastani	Unclear	Low risk	Low risk	Low risk	Low risk	Unknown
Abadi	Unclear	Unclear	Low risk	Low risk	High risk	Unknown
Zick	Unclear	Low risk	Unclear	High risk	Low risk	Unknown
Mohaddes Ardabili	Low risk	Unclear	Unclear	Low risk	Low risk	Unknown
Dehghanmehr	Unclear	Low risk	Low risk	Low risk	High risk	Unknown
Pouy	Unclear	High risk	Unclear	Low risk	High risk	Unknown
Samadi	Low risk	Low risk	Low risk	High risk	Low risk	Unknown
Horiuchi	Unclear	High risk	High risk	Low risk	Low risk	Unknown
Kanza Gul	Unclear	Low risk	Low risk	Low risk	Low risk	Unknown
Vasokolaei	Low risk	Low risk	Low risk	Unclear	Low risk	Unknown
Mansoorzadeh	Unclear	Unclear	Low risk	Low risk	Low risk	Unknown
Genc	Unclear	Unclear	Unclear	Low risk	Low risk	Unknown
Mącznik	Unclear	High risk	High risk	Low risk	Low risk	Unknown
Sharifi Rizi	Low risk	Unclear	Low risk	Low risk	Low risk	Unknown
Rarani	Low risk	Unclear	Low risk	Low risk	Low risk	Unknown
Dharwal	Low risk	Unclear	Unclear	Low risk	Low risk	Unknown
Avisa	Low risk	Low risk	Low risk	Low risk	Low risk	Unknown
Borji	Low risk	Low risk	Low risk	Low risk	Low risk	Unknown
Kuo	Low risk	Low risk	Low risk	Low risk	Low risk	Unknown
Kafaei-Atrian	Low risk	Low risk	Low risk	Low risk	Low risk	Unknown
Moradi	Unclear	Unclear	High risk	Low risk	Low risk	Unknown
Tseng	Low risk	Low risk	Low risk	Low risk	Low risk	Unknown
Lin	Low risk	Low risk	Low risk	High risk	Low risk	Unknown
Luo	Low risk	Low risk	Low risk	Low risk	Low risk	Unknown
Bang	Low risk	Low risk	Low risk	Low risk	Low risk	Unknown
Sangani	Unclear	Unclear	Unclear	Low risk	Low risk	Unknown
Lee	Unclear	Low risk	Low risk	Low risk	Low risk	Unknown
Abd Elgwad Ali	Unclear	Low risk	Low risk	Low risk	Low risk	Unknown
Bal	Low risk	Low risk	Low risk	Low risk	Low risk	Unknown
Derya Ister	Unclear	Low risk	Low risk	High risk	Low risk	Unknown
Cai	Low risk	Low risk	Low risk	Low risk	Low risk	Unknown
Masoudi	Unclear	Unclear	Low risk	Low risk	Low risk	Unknown
Cho	Low risk	Unclear	Low risk	Low risk	Low risk	Unknown
Consolação Soares	Low risk	Low risk	Unclear	High risk	Low risk	Unknown
Yanik	Low risk	Low risk	Unclear	Low risk	Low risk	Unknown

### Meta analysis results

#### STAI scale

**Figure 3:**
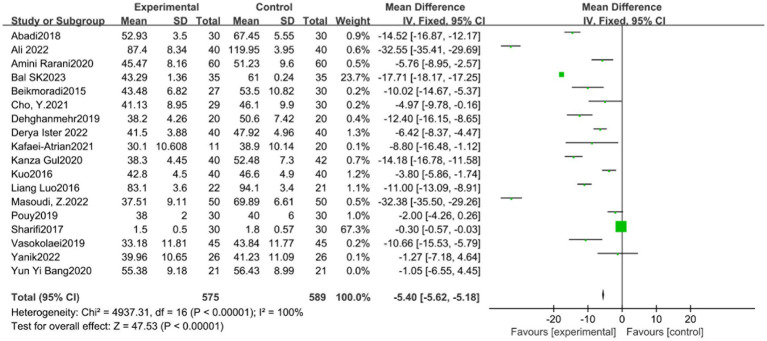
STAI meta analysis forest map.

**Figure 4:**
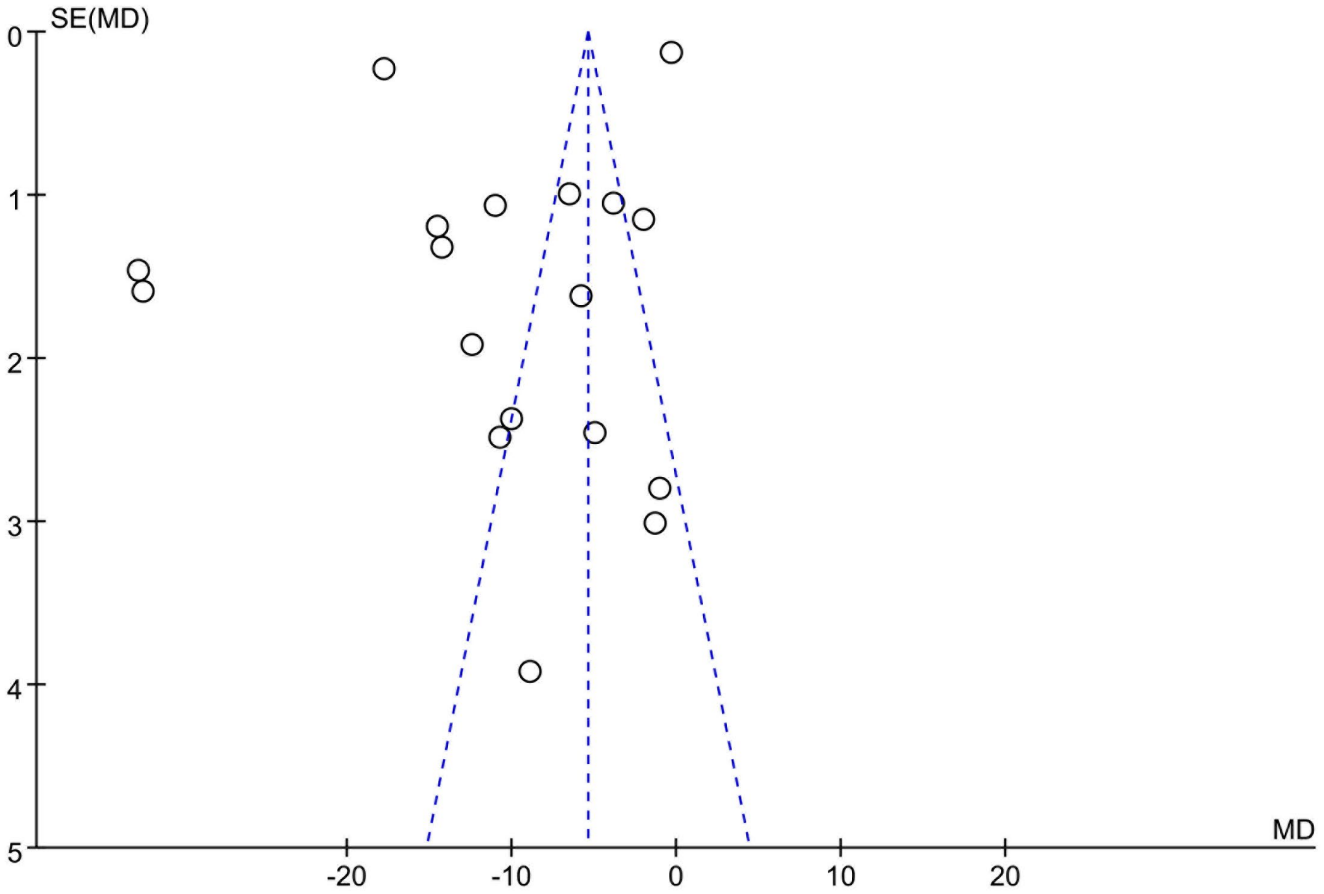
STAI scale assessment funnel plot.

The STAI scale included 18 studies. The experimental group (596 cases) and the control group (610 cases) demonstrated significant heterogeneity among the studies (*p* < 0.00001, *I*^2^ = 100%), as shown in Figure 3. Sensitivity analysis showed that the study of Sharifi Rizi et al. (6, 15, 17, 40) may be the primary source of heterogeneity. Heterogeneity among studies decreased after the exclusion of this reference (*p* < 0.00001, *I*^2^ = 100%). Given the source of heterogeneity was related to the differences in the study subjects, subgroup analysis was conducted according to the characteristics of the study subjects (group 1 patients and group 2 non-patients). The analysis results showed significant difference between the experimental group (SMD = −5.39, 95% CI: −5.61 to −5.17, *p* < 0.01) and the control group (SMD = −5.40, 95% CI: −5.62 to −5.18, *p* < 0.01) in both subgroups. However, the funnel plot (Figure 4) suggested potential publication bias, thereby reducing the credibility of the conclusion.

#### VAS scale

**Figure 5:**
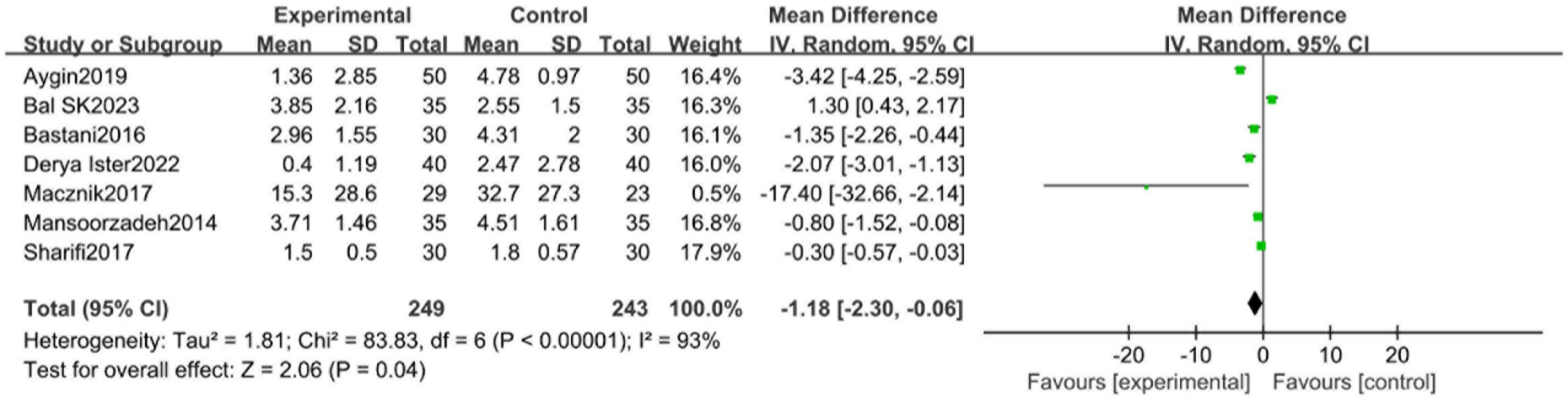
VAS meta analysis forest map.

In assessing the Visual Analogue Scale (VAS), a total of 6 studies were incorporated. These studies, divided into an experimental group (249 participants) and a control group (243 participants), demonstrated significant heterogeneity (*p* < 0.00001, *I*^2^ = 93%), as illustrated in Figure 5. Sensitivity analysis identified the study by Aygin et al. (8) as a potential source of heterogeneity. Upon its exclusion, the heterogeneity among studies decreased (*p* < 0.00001, *I*^2^ = 87%).

#### SAS scale

**Figure 6:**
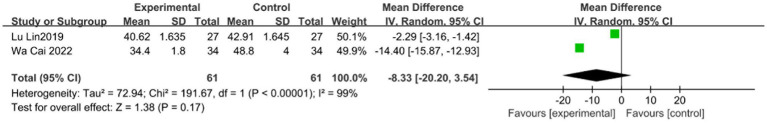
SAS meta analysis forest map.

Two studies were included in the evaluation of the Self-Rating Anxiety Scale (SAS). Both the experimental group (61 participants) and the control group (61 participants) displayed significant heterogeneity among the studies (*p* < 0.00001, *I*^2^ = 99%), as depicted in Figure 6.

#### DASS scale

**Figure 7:**
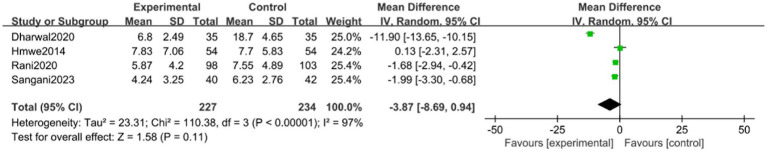
DASS meta analysis forest map.

Four studies were included in the evaluation of the Depression Anxiety and Stress Scale (DASS-21/DASS-42). With 227 participants in both the experimental group and the control group, there was significant heterogeneity across the studies (*p* < 0.00001, *I*^2^ = 97%), as indicated in Figure 7. Sensitivity analysis indicated the study of Dharwal et al. (41) as a possible cause of heterogeneity. Once this reference was excluded, heterogeneity among studies decreased (*p* < 0.00001, *I*^2^ = 12%).

#### BAI scale

**Figure 8:**
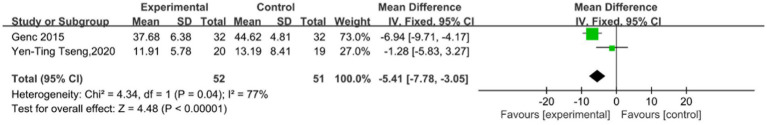
BAI meta analysis forest map.

In assessing the Beck Anxiety Inventory (BAI), two studies were included, encompassing an experimental group (61 participants) and a control group (52 participants). These studies showed heterogeneity (*p* < 0.00001, *I*^2^ = 77%), as shown in Figure 8.

Similarly, VAS, SAS, DASS, and BAI scales included studies demonstrating significant heterogeneity (*p* < 0.00001, *I*^2^ > 75% for all scales). Sensitivity analyses and exclusion of certain studies decreased heterogeneity in each scale. Nine studies evaluated anxiety using HADS (12), BSPAS (13), FAS (16), POMS-J (17), MCDAS (24), mYPAS (25), SAQ (28), RTA (42), and VPTm (43). They were excluded because they were not representative and had fewer than two studies included, making them unsuitable for bias risk assessment.

### Sensitivity analysis and publication bias

**Figure 9:**
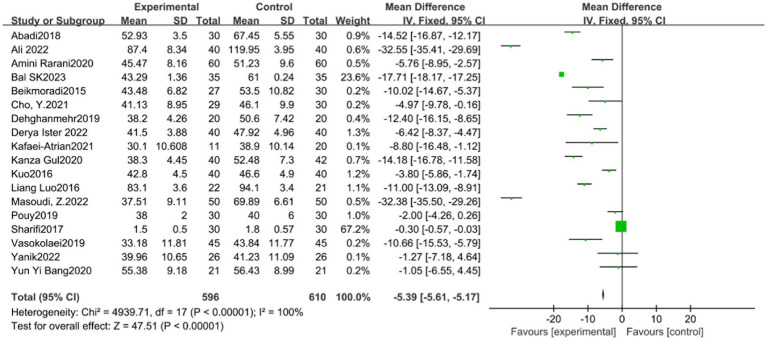
STAI sensitivity analysis chart.

Sensitivity analysis suggested that the main sources of heterogeneity came from the studies of Aygin and Şen (8), Rani et al. (9) and Sharifi Rizi et al. (22), as *I*^2^ decreased to 51% after their removal (Figure 9). The funnel plot of changes in anxiety levels was symmetric, indicating no detectable publication bias (Figure 4).

### Subgroup analysis

**Figure 10:**
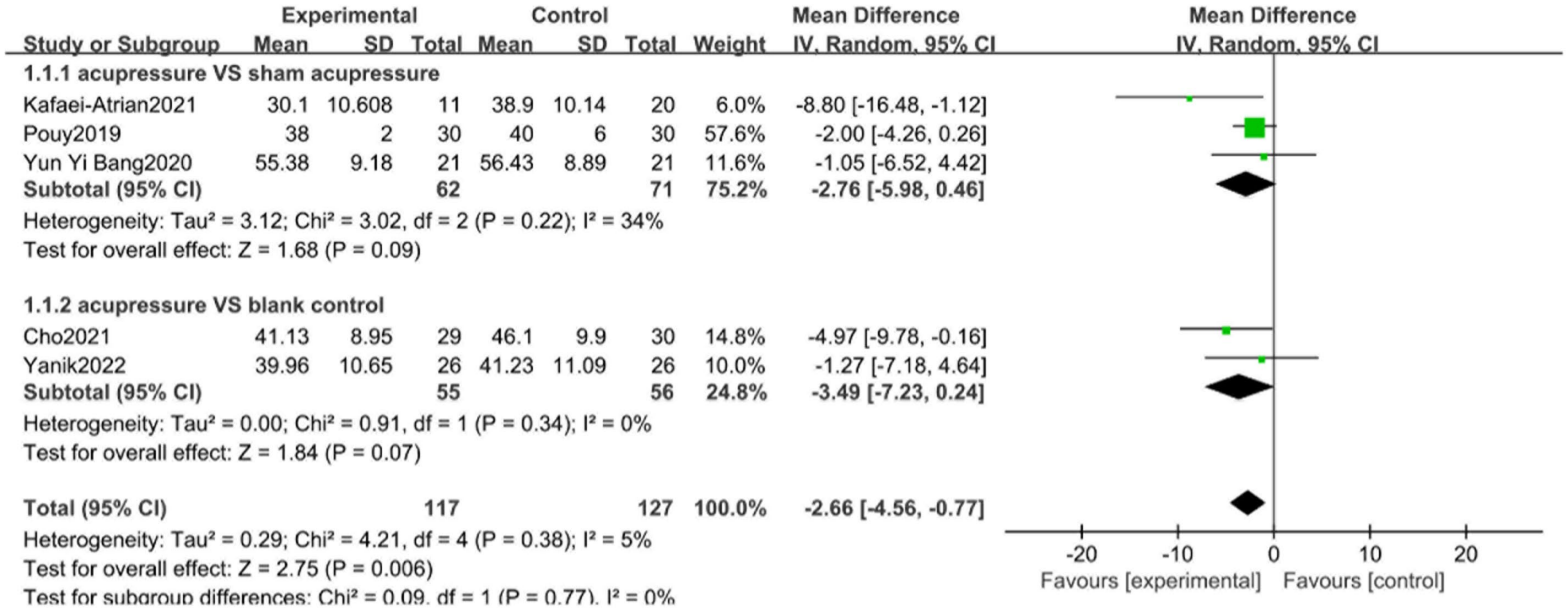
SATI subgroup analysis chart.

Subgroup analysis was conducted to verify whether different interventions in the control group would influence changes in anxiety levels. According to the STAI subgroup analysis, the therapeutic effect of the acupressure group on anxiety levels was higher than that of the sham intervention group and blank control group, with low heterogeneity between groups (*I*^2^ = 0%, *p* = 0.77) (Figure 10). Three trials (15, 27, 32) including 133 patients using random effects models demonstrated that acupressure was more effective than sham acupressure in improving changes in anxiety levels (SMD −2.76, 95% CI: −5.98 to 0.46, *p* = 0.09). Two trials (39, 44) compared acupressure and blank controls in assessing the effect on changes in anxiety levels, but results were significantly different (SMD −3.49, 95% CI: −7.23 to 0.24, *p* = 0.07).

## Discussion

**Figure 11:**
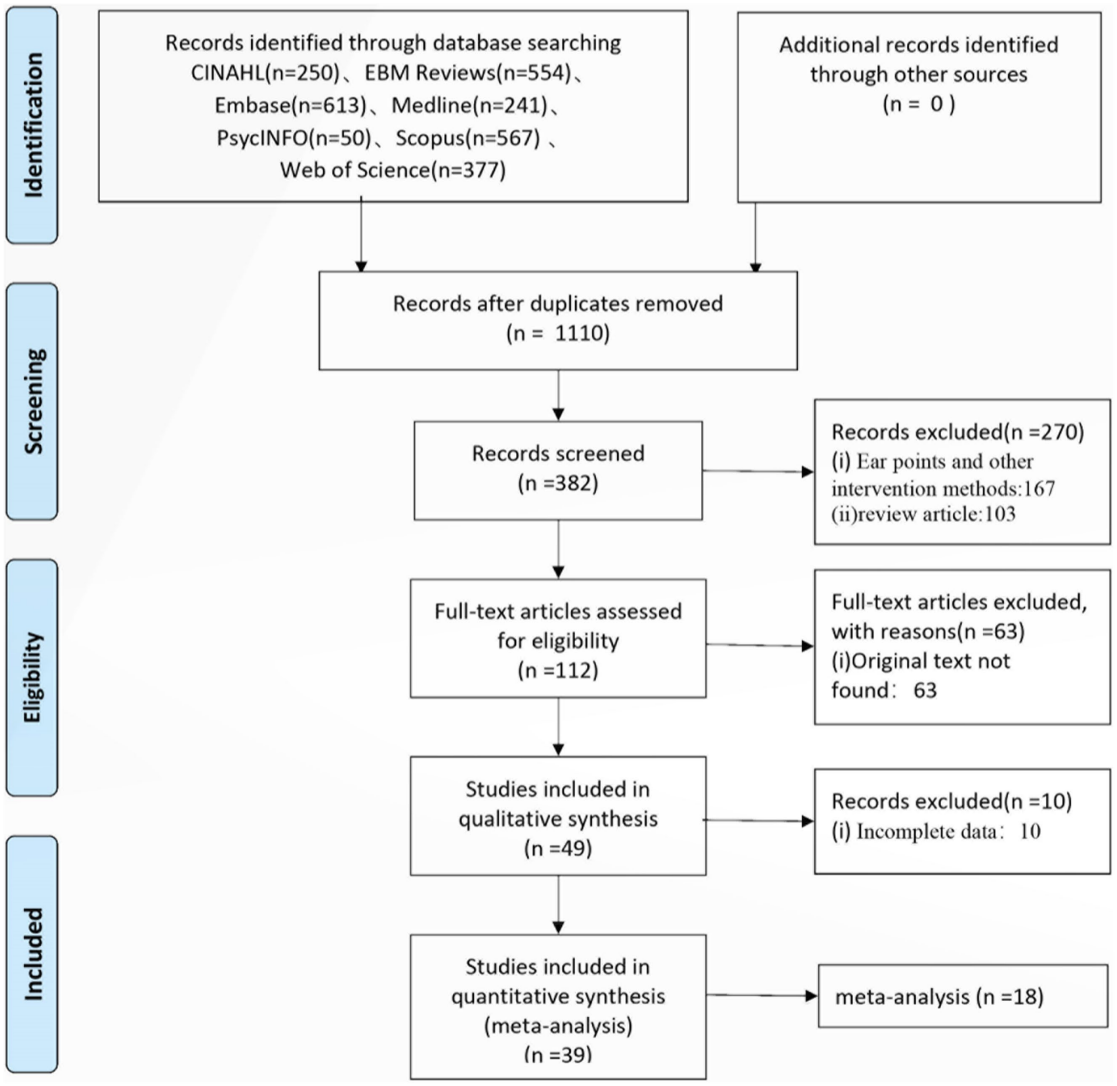
PRISMA 2009 flow diagram.

Of the total of 3,395 studies reviewed, including 39 randomized controlled trials, 103 systematic reviews and 18 meta-analysis (Figure 11), acupressure was found to be an effective intervention for anxiety. Sham acupressure and blank controls are typically designed to help mitigate bias when assessing acupressure’s specific effects. According to the results of subgroup analysis, the acupressure group displayed a higher therapeutic effect on anxiety levels than the sham intervention group and the blank control group.

### Current treatments on anxiety

Anxiety symptoms typically encompass both physical and psychological manifestations such as excessive worry, fatigue, muscle or jaw tension, sleep difficulties, increased heart rate, and sweating. Severe anxiety may also induce symptoms like nausea, headaches, and lack of concentration. To alleviate these symptoms, many individuals resort to use medication including benzodiazepines (45), non-benzodiazepines and anti-anxiety antidepressants, which can potentially lead to side effects and dependency (46, 47). Psychotherapy, through physical and verbal communication, can establish a positive doctor-patient relationship, guiding and aiding patients to alter detrimental behavior habits, cognitive concepts and psychological states (48).

Physical therapy methods such as massage, acupuncture, transcranial magnetic stimulation treatment can also alleviate anxiety and soothe the body and mind. Studies suggest that acupuncture and electroacupuncture can effectively treat anxiety either independently or as adjuncts to pharmacological therapy (49). Acupuncture therapy may reduce preoperative patient anxiety (50). rTMS presents as a feasible therapeutic option for high-prevalence neuropsychiatric dysfunctions and contributes to our understanding of pathological and neuropsychological adaptation processes (51).

Other treatments include practices like yoga, jogging, tai chi and other aerobic exercises, as well as distraction by studying, listening to music, painting, etc., which can all contribute to treating anxiety disorders. Meta-analyses and systematic reviews have shown that these interventions can improve symptoms of depression and anxiety disorders (52).

### Effects of acupressure on anxiety

Acupressure, an ancient nonpharmacological technique used for symptom management, involves the application of steady, gentle pressure on one or more of the body’s 365 energy points across 12 meridians, thereby creating balance and releasing energy. Simple to administer and requiring no instruments, acupressure is suitable for various demographics, from children to the elderly, and can aid in managing clinical symptoms such as dyspnea, pain, insomnia, nausea and vomiting. From a scientific perspective, acupressure aims to influence the sympathetic and parasympathetic systems through pressure application, thereby releasing neurotransmitters and mediators (53), and ultimately relieving anxiety. Studies indicate that acupressure is effective for generalized anxiety disorder and provides lasting benefits (54).

### Mechanism of acupressure on anxiety

The etiology and pathogenesis of anxiety disorder are complex, believed to involve a variety of factors including genetics, neurobiochemistry, neuroimaging, sex hormones, constitution and other reasons (55–57). Contemporary studies propose that the pathogenesis of anxiety disorder primarily encompasses neurotransmitter hypothesis and neuroendocrine dysfunction hypothesis, specifically the serotonin system, the hypothalamic-pituitary-adrenal (HPA) axis (58), and hypothalamic-pituitary-gonadal (HPG) axis activity (59).

The potential mechanism of acupressure and its treatment of anxiety remains unclear. Most existing literature focuses on general clinical summaries or efficacy observations, and few studies delving into basic research. Acupressure is believed to stimulate specific points on the body, regulating human function, balancing yin and yang, relieving fatigue, and preventing disease (59). The temple can regulate the autonomic nervous system, compensate the heart, and calm the mind. The product of the three yin, involving the liver, spleen, kidney meridians, can have soothing effects. Acupressure massage, grounded in meridian acuity theology, uses massage as the main treatment method, serving as a preventative and therapeutic approach (46). The primary physiological reasons involved in the massage stimulation process might include the stimulation of serotonin to alleviate pain or emotional discomfort and/or expected psychological responses to stress or perceived environmental threats. Acupressure massage has been shown to reduce heart rate, pulse rate and blood pressure by suppressing the sympathetic nerve and activating the parasympathetic nerve, thus relieving anxiety (60).

Mechanism of acupressure on anxiety recent advances in animal models of anxiety, have greatly enhanced our understanding of the potential mechanisms of acupoint therapy in treating anxiety disorders. Four potential mechanisms have been proposed: it may be related to the up-regulation of atrial natriuretic peptide (ANP) expression and downregulation of C-type natriuretic peptide (CNP) expression in the peripheral adrenal medulla, which in turn inhibits the release of corticosterone (CORT) and the activity of hypothalamic-pituitary-adrenal axis (HPA) (61). Acupressure may inhibit the elevation of amygdala-like norepinephrine (NE) and 3-methoxy-4-hydroxyphenylethylene glycol (MHPG), induced by acute restraint stress (ARS), and prevent the enhancement of tyrosine hydroxylase protein and mRNA expression in the central nucleus of amygdala (CeA) (62). Acupressure can also significantly reduced depressive-like behaviors caused by chronic unpredictable stress (CUS), and the expression of certain NLRP3 and mature IL-1b (63). Lastly, following Tuina, anxiety-like behaviors were efficiently reduced, and the hyperactivity of the HPA axis was efficiently inhibited, along with enhanced GR expression in the hippocampus and lung (64).

### Intervention population

Although the effectiveness of acupressure in relieving STAI has been confirmed, the heterogeneity is relatively large. This could be attributed to the wide range of research subjects included in this study, encompassing healthy individuals such as expectant mothers, parents of children awaiting surgery, military personnel, and college students, as well as patients with various pre- and post-surgery, cancer, hemodialysis, burns, sports injuries. However, subgroup analysis did not indicate a decrease in heterogeneity. This could be due to factors such as the choice of acupuncture points and massage duration.

### Strengths and limitations

The global spread of COVID-19 has triggered numerous social issues related to health, economy, and society, all of which are important factors contributing to anxiety. To our knowledge, acupressure is a viable method for relieving anxiety. Amid widespread pandemic widespread concerns, acupressure serves massage as a practical treatment strategy with numerous advantages: it is easily implemented, cost-effective, safe, devoid of toxic side effects, and easy acceptance by people readily accepted by different age groups and populations.

Our study benefits from several strengths. Firstly, we focused our review on the effect of acupressure as a standalone treatment, excluding studies involving mixed therapies, and conducted a subgroup study of sham acupressure or blank control in the control group to verify whether acupressure’s effectiveness in treating anxiety. Secondly, our review included 39 RCTS with larger sample sizes and a variety of acupressure points. Compared with previous studies, our study included patients of varying ages, encompassing both diseased and non-diseased populations, thus providing strong evidence supporting the hypothesis that acupressure is effective in treating anxiety. Thirdly, the included studies were conducted at multiple locations and in different countries, covering a diverse range of ethnicities and cultures, potentially reducing selection bias and improving external validity. Fourthly, we conducted sensitivity analysis and funnel plot, indicating that the meta-analysis was stable, robust, and free from publication bias. Lastly, most of the studies were longitudinal, with one having a follow-up period of 1 year, which lends further support to the clinical practice of acupressure in the treatment of patients with anxiety.

However, there are limitations to consider when interpreting these results. This review only included RCTs, thereby excluding observational and non-randomized studies. Most of the included studies did not feature follow-up evaluations, preventing a comprehensive meta-analysis of acupressure’s long-term effects. The overall quality of the studies was low, particularly concerning allocation concealment and participant and personnel blindness. Furthermore, this review only included English-language, excluding potential insights from non-English sources.

### Implications for further research

Acupressure demonstrates promising application prospects. However, a unified and standardized acupoint selection plan is lacking, and there are limited studies conducting in-depth analyses from an anti-anxiety mechanism perspective. Future research would benefit from a more standardized approach to acupressure point selection, alongside more extensive studies examining the anti-anxiety mechanisms involved.

## Conclusion

Acupressure has a beneficial overall effect of acupressure in relieving anxiety. Considering the increasing incidence of anxiety caused by long COVID, acupressure represents an ideal treatment strategy. Its unique convenience and cost-effectiveness can expand its application and provide relief to a larger population suffering from anxiety. Further rigorous research focusing on the mechanisms behind its anti-anxiety effects, as well as well-designed studies to reinforce these findings, are necessary.

## Author contributions

ZP: Formal analysis, Writing – review & editing. YZ: Writing – original draft. ZY: Writing – original draft, Methodology. HZ: Writing – review & editing. ZL: Writing – original draft, Data curation. MX: Writing – review & editing, Funding acquisition, Resources. SC: Writing – review & editing, Funding acquisition, Resources. RL: Writing – review & editing, Project administration, Supervision.
